# Single-Cell RNA Sequencing Identifies Crucial Genes Influencing the Polarization of Tumor-Associated Macrophages in Liver Cancer

**DOI:** 10.1155/2024/7263358

**Published:** 2024-05-25

**Authors:** Kedong Xu, Mingyi Dong, Zhengqiang Wu, Linfei Luo, Fei Xie, Fan Li, Hongyan Huang, Fenfen Wang, Xiaofeng Xiong, Zhili Wen

**Affiliations:** Department of Gastroenterology The Second Affiliated Hospital of Nanchang University, Nanchang, Jiangxi, China

## Abstract

**Background:**

In the context of hepatocellular carcinoma (HCC), tumor-associated macrophages (TAMs) are pivotal for the immunosuppressive nature of the tumor microenvironment (TME). This investigation delves into the functional transformations of TAMs within the TME by leveraging single-cell transcriptomics to pinpoint critical genes influencing TAM subset polarization.

**Methods:**

We procured single-cell and bulk transcriptomic data from the Gene Expression Omnibus (GEO) and The Cancer Genome Atlas (TCGA), implementing quality assurance, dimensional reduction, clustering, and annotation on the single-cell sequencing data. To examine cellular interactions, CellChat was utilized, while single-cell regulatory network inference and clustering (SCENIC) was applied to deduce transcription factors (TFs) and their associated targets. Through gene enrichment, survival, and immune infiltration correlation analyses, we sought to pinpoint and validate influential genes. A TAM model under HCC conditions was then established to confirm the expression levels of these key genes.

**Results:**

Our analysis encompassed 74,742 cells and 23,110 genes. Through postdimensional reduction and clustering, we identified seven distinct cell types and nine TAM subtypes. Analysis via CellChat highlighted a predominance of M2-phenotype-inclined TAM subsets within the tumor's core. SCENIC pinpointed the transcription factor PRDM1 and its target genes as pivotal in this region. Further analysis indicated these genes' involvement in macrophage polarization. Employing trajectory analysis, survival analysis, and immune infiltration correlation, we scrutinized and validated genes likely directing M2 polarization. Experimental validation confirmed PRDM1's heightened expression in TAMs conditioned by HCC.

**Conclusions:**

Our findings suggest the PRDM1 gene is a key regulator of M2 macrophage polarization, contributing to the immunosuppressive TME in HCC.

## 1. Introduction

Hepatocellular carcinoma (HCC) stands as the primary histological variant of liver cancer, occupying the sixth position among the most prevalent malignant tumors worldwide and ranking third in cancer-related mortality [1]. Despite witnessing a steady decrease in both incidence and mortality rates of HCC in China, this nation continues to shoulder the most substantial burden of this disease. HCC's risk factors encompass viral hepatitis, nonalcoholic fatty liver disease, and alcoholic liver disease [2]. Often, HCC's stealthy progression leads to late-stage diagnoses, where treatment options remain scarce, culminating in a median survival time of merely 6-10 months across various HCC etiologies [3, 4]. Although the advent of immune checkpoint inhibitors has introduced new hope in HCC treatment, their effectiveness is limited to patients with sufficient effector T cell presence, showing a response rate below 30% when used as monotherapy [5]. Given HCC's inherent inflammatory nature, it presents a compelling case for further exploration within the immunotherapy realm.

The liver cancer tumor microenvironment is intricately composed of various cellular entities, including hepatoma cells, tumor-associated macrophages (TAMs), T cells, B cells, and NK cells, alongside stromal cells like tumor-associated endothelial cells (TEC) and cancer-associated fibroblasts (CAF) [6]. TAMs, constituting a significant portion of immune cells within the tumor microenvironment—ranging from 20% to 50% of the total immune cell infiltration—play a pivotal role [7, 8]. The traditional dichotomy of TAMs categorizes them into M1-like TAMs, which hinder tumor progression, and M2-like TAMs, which facilitate tumor growth, with the latter being more prevalent [9, 10]. TAMs are characterized by their remarkable plasticity, capable of altering their functional phenotypes in response to changes in the tumor microenvironment, thereby contributing to HCC's heterogeneity and influencing treatment outcomes [11]. This study is aimed at delving into the functional dynamics of macrophage subpopulations within the tumor microenvironment using single-cell transcriptome sequencing for bioinformatics analysis, with a focus on identifying pivotal genes that govern macrophage polarization.

## 2. Materials and Methods

### 2.1. Data Collection

For our study, we sourced hepatocellular carcinoma (HCC) single-cell transcriptomic datasets (GSE189903) from the Gene Expression Omnibus (GEO; GEO database), focusing on samples from four patients [12]. These encompassed three key areas: the tumor core, tumor border, and adjacent normal tissues, resulting in a collection from five distinct sampling sites with a total of 20 sequencing samples. We further augmented our dataset with bulk transcriptome data from The Cancer Genome Atlas (TCGA, TCGA portal), utilizing the “TCGAbiolinks” package for comprehensive analysis [13].

### 2.2. Single-Cell Transcriptome Data Processing and Analysis

The single-cell RNA sequencing (scRNA-seq) data underwent processing and normalization using the “Seurat” package, beginning with stringent quality control measures [14]. We assessed cell viability based on gene expression counts and mitochondrial gene content, excluding cells deemed low-quality or dead (criteria: genes expressed in at least three cells, excluding cells with <200 or >6000 gene counts and those with >20% mitochondrial gene content). Postcleaning, we leveraged the “FindVariableFeatures” function for identifying variable features and applied principal component analysis (PCA) for dimensionality reduction. To cluster data, we used uniform manifold approximation and projection (UMAP) on the leading 20 principal components (PCs), employing the “FindAllMarkers” function for marker gene identification. Cell type annotation was supported by the CellMarker database (CellMarker) and existing literature for accurate categorization [15].

### 2.3. Cell Proportion Statistics

Given the prognostic significance of cell types and proportions within the tumor microenvironment, we computed and compared these metrics across different tissue regions to elucidate the potential roles of various cells within the tumor ecosystem.

### 2.4. TAM Subset Analysis

Acknowledging the heterogeneous nature of tumor-associated macrophages (TAMs), we conducted a secondary clustering of macrophage data. This enabled us to examine the distribution and expression profiles of signature genes within each macrophage subset, offering insights into their specific functional roles.

### 2.5. Cell Interaction Analysis

To decode the intricacies of cell-to-cell communication within the tumor microenvironment, we employed the “cellchat” package, which facilitates the quantitative analysis of interaction networks based on ligand-receptor-cofactor databases derived from scRNA-seq data [16]. By following the CellChat standard workflow, we quantified and characterized the interaction dynamics, strengths, and pathways between different cell populations, with a particular focus on TAM subsets, to better understand their influence on the tumor microenvironment.

### 2.6. Gene Regulatory Network Analysis

SCENIC, a method grounded in coexpression and motif analysis, serves to derive gene regulatory networks (GRNs) from single-cell transcriptomic data, facilitating the identification of distinct cellular states [17]. We initiate our analysis with standardized single-cell transcriptome expression data, sourcing human genome reference data from the cisTarget databases (https://resources.aertslab.org/cistarget/) including specific datasets for comprehensive motif analysis. Additionally, a transcription factor list is retrieved from GitHub (https://github.com/aertslab/pySCENIC/tree/master/resources) to ensure a focused analysis using pySCENIC [18]. The procedure encompasses three critical steps: (1) inferring coexpression modules between transcription factors and target genes via GRNBoost; (2) employing RcisTarget for *cis*-regulatory motif analysis within each module, thus filtering out indirect target genes and preserving those modules enriched in TF motifs; and (3) utilizing AUCell to evaluate the regulon activity across individual cells, forming a matrix to depict cell regulon activities.

### 2.7. Scissor Analysis

Recognizing cell subpopulations pivotal to disease progression, metastasis, and survival outcomes is crucial. The “Scissor” package offers a streamlined approach to pinpoint cell subpopulations significantly influencing phenotypic distinctions from single-cell datasets, connecting cell clusters with specific phenotypes directly [19]. By integrating single-cell expression data with TCGA-LIHC data linked to clinical prognostic indicators, we identify macrophage subpopulations closely associated with poor prognosis in liver carcinoma, employing a precise parameter setting for robust analysis.

### 2.8. Trajectory Analysis

Monocle2 adopts an unsupervised learning approach through the DDRTree algorithm for reversed graph embedding, enabling the construction of cellular trajectories. This method elucidates cellular evolution and differentiation at the single-cell level, highlighting gene regulation's role in determining cell fate [20]. The identification of highly variable genes for trajectory construction and differential expression analysis across pseudotemporal stages are crucial for understanding macrophage polarization dynamics.

### 2.9. Enrichment Analysis

Focusing on the PRDM1 regulon, which exhibits notable regulon activity and specificity scores, we analyze its comprising transcription factor PRDM1 and 644 predicted targets. Intersection with differentially expressed genes from trajectory analysis yields 101 significant genes, categorized into three clusters for functional enrichment analysis. This step aims to unravel the biological implications of these clusters on cellular functions.

### 2.10. TCGA Data Analysis

UALCAN (http://ualcan.path.uab.edu) and TIMER2.0 (http://timer.cistrome.org/) serve as our analytical tools for probing TCGA data, facilitating the exploration of gene expression disparities, clinical outcomes, and the correlation between gene expression and immune cell infiltration within TCGA-LIHC [21, 22]. This dual approach allows for a comprehensive examination of the prognostic significance of 41 genes in cluster 1 and the validation of their association with macrophage infiltration levels, underscoring the interconnectedness between gene expression profiles and immune dynamics in the tumor microenvironment.

### 2.11. Cell Culture and Coculture System

Human hepatocellular carcinoma cell lines (Huh 7 and MHCC-LM3) and the human monocytic cell line (THP-1) were acquired from the Chinese Academy of Sciences (Shanghai, China). The HCC cell lines were propagated in Dulbecco's Modified Eagle Medium (DMEM) supplemented with 10% fetal bovine serum (FBS). THP-1 cells were maintained in RPMI-1640 medium enriched with 10% FBS, 100 U/mL penicillin, and 100 *μ*g/mL streptomycin. To differentiate THP-1 cells into M0 macrophages, they were treated with 100 ng/mL phorbol 12-myristate 13-acetate (PMA) for 24 hours. All cultures were incubated at 37°C in a 5% CO₂ atmosphere. A transwell insert system (0.4 *μ*m pore size, Corning) was established for coculture, placing 1 × 10^5^ liver cancer cells in each insert above 1 × 10^6^ M0 macrophages in the lower chamber to model tumor cell and macrophage interactions. After 72 hours of coculture, cells from the lower chamber were harvested for analysis of M1 and M2 macrophage markers, along with PRDM1 expression via RT-qPCR and Western blot.

### 2.12. RNA Extraction and Real-Time Reverse Transcription PCR

RNA was extracted using a TRIzol reagent, with its concentration measured subsequently. cDNA synthesis was carried out using the FastKing cDNA First Strand Synthesis Kit (Tiangen Biotech, Beijing, China). A 20 *μ*L reaction system with SuperReal PreMix Plus (SYBR Green) (Tiangen Biotech, Beijing, China) was utilized to quantify the expression of the panmacrophage marker CD68, M1 macrophage markers CD86 and iNOS, M2 macrophage markers MRC1 and CD163, and PRDM1. Primer sequences for these genes were carefully selected for specificity. GAPDH served as the normalization control, employing the 2^−ΔΔCT^ method for relative mRNA expression analysis.

### 2.13. Protein Extraction and Western Blot

Protein was extracted using RIPA buffer containing 1% PMSF. The BCA protein assay kit determined protein concentrations, with 40 *μ*g of protein per sample separated by 10% SDS-PAGE and transferred onto PVDF membranes. After blocking with 5% nonfat milk, the membranes were incubated overnight at 4°C with primary antibodies against CD163, PRDM1, and *β*-actin. Secondary antibody incubation was followed for 1 hour at room temperature. Chemiluminescent detection and ImageJ software were used for band visualization and intensity quantification.

### 2.14. Statistical Analysis

Statistical evaluations were executed using R software (version 4.2.2) and GraphPad Prism (version 9.0), with categorical data expressed as counts and percentages and continuous data as mean ± SE or median. Differences between groups were assessed via *t*-tests or ANOVA, considering *p* values < 0.05 as statistically significant.

## 3. Result

### 3.1. Cell Clustering, Annotation, and Identification of Macrophage Subsets

**Figure 1:**
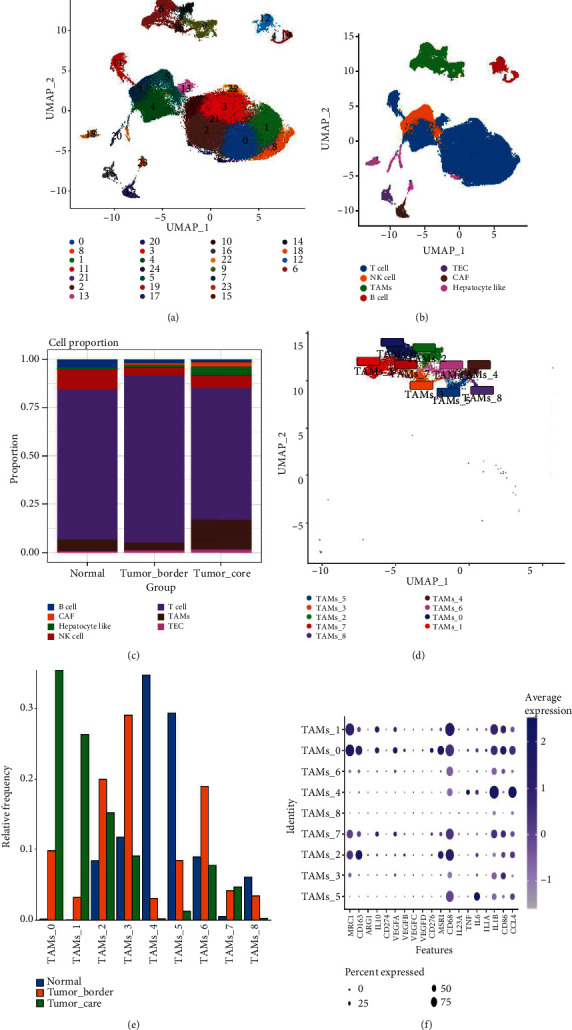
Identification of cell clusters and annotation. (a) 74742 cells are clustered into 25 clusters. (b) Cell type annotation. (c) Cell proportion statistics. (d) Macrophage subset analysis. (e) Macrophage subset distribution characteristics. (f) Dot plot of macrophage subset-specific markers.

Our meticulous quality control measures yielded a dataset comprising 74,742 cell samples, covering 23,110 genes. Utilizing PCA for dimensionality reduction and selecting the top 20 principal components for subsequent UMAP analysis, we identified 25 distinct cell clusters (Figure 1(a)). Annotation of these clusters allowed us to classify seven cell types within the samples: T cells, NK cells, tumor-associated macrophages (TAMs), B cells, tumor-associated endothelial cells (TEC), cancer-associated fibroblasts (CAF), and hepatocyte-like cells (Figure 1(b)).

An in-depth analysis of cell proportions across three different tissue regions revealed a notable increase in macrophage presence within the tumor core, constituting about 15% of total cells—making them the second most prevalent cell type following T cells. Conversely, the representation of T cells, NK cells, B cells, and other immune cell types markedly decreased in the tumor core. Further refinement through secondary clustering of macrophages elucidated nine distinct macrophage subpopulations (Figure 1(c)). Within the tumor core, the TAMs_0, TAMs_1, TAMs_2, and TAMs_7 subpopulations predominantly expressed M2-like macrophage markers (e.g., MRC1, IL10, and CD163), indicating an inclination towards the M2 phenotype. Conversely, the TAMs_4 and TAMs_5 subpopulations, which leaned towards expressing M1-like macrophage markers, were mainly found in adjacent normal tissues. The TAMs_3 and TAMs_6 subpopulations, which were most abundant at the tumor margin, showcased intermediate phenotypes between M1 and M2-like macrophages (Figures 1(d), 1(e), and 1(f)).

### 3.2. Insights from CellChat

**Figure 2:**
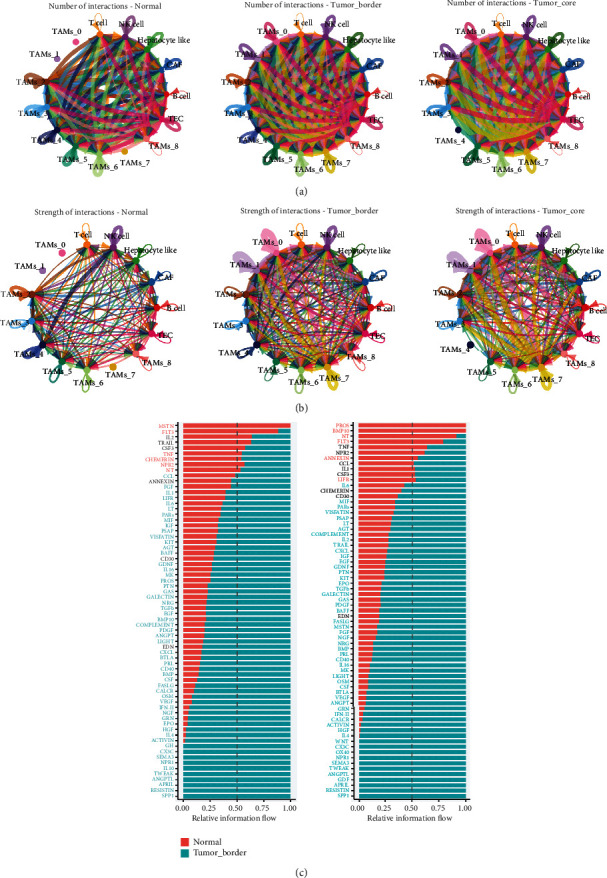
Strength and pathways involved in cell-cell interactions from three regions in the tumor core, tumor border, and adjacent normal tissue. (a) The number of interactions between cells. (b) The strength of interactions between cells. (c) The communication probability of pathways from normal to tumor border and normal to tumor core.

CellChat analysis revealed that intercellular interactions within the tumor tissues were significantly more intense and numerous compared to those in adjacent nontumor tissues. Specifically, in the tumor core, TAMs_0, TAMs_1, and TAMs_7 subpopulations exhibited a marked increase in signal transmission, both received and emitted. This increase was also observed in the signal output from tumor cells, suggesting that interactions between tumor cells and TAM subpopulations (TAMs_0, TAMs_1, and TAMs_7) may drive macrophage polarization towards the M2 phenotype within the tumor core (Figures 2(a) and 2(b)).

Moreover, signaling pathways such as SPP1, APRIL (TNFSF13), and IL4 were notably enhanced in the tumor region. These pathways are critically linked to the promotion of M2 macrophage polarization, underscoring their potential role in shaping the immunosuppressive microenvironment that characterizes tumor progression (Figure 2(c)) [23–25].

### 3.3. Gene Regulatory Networks and Prognostic Features of Macrophage Subsets

**Figure 3:**
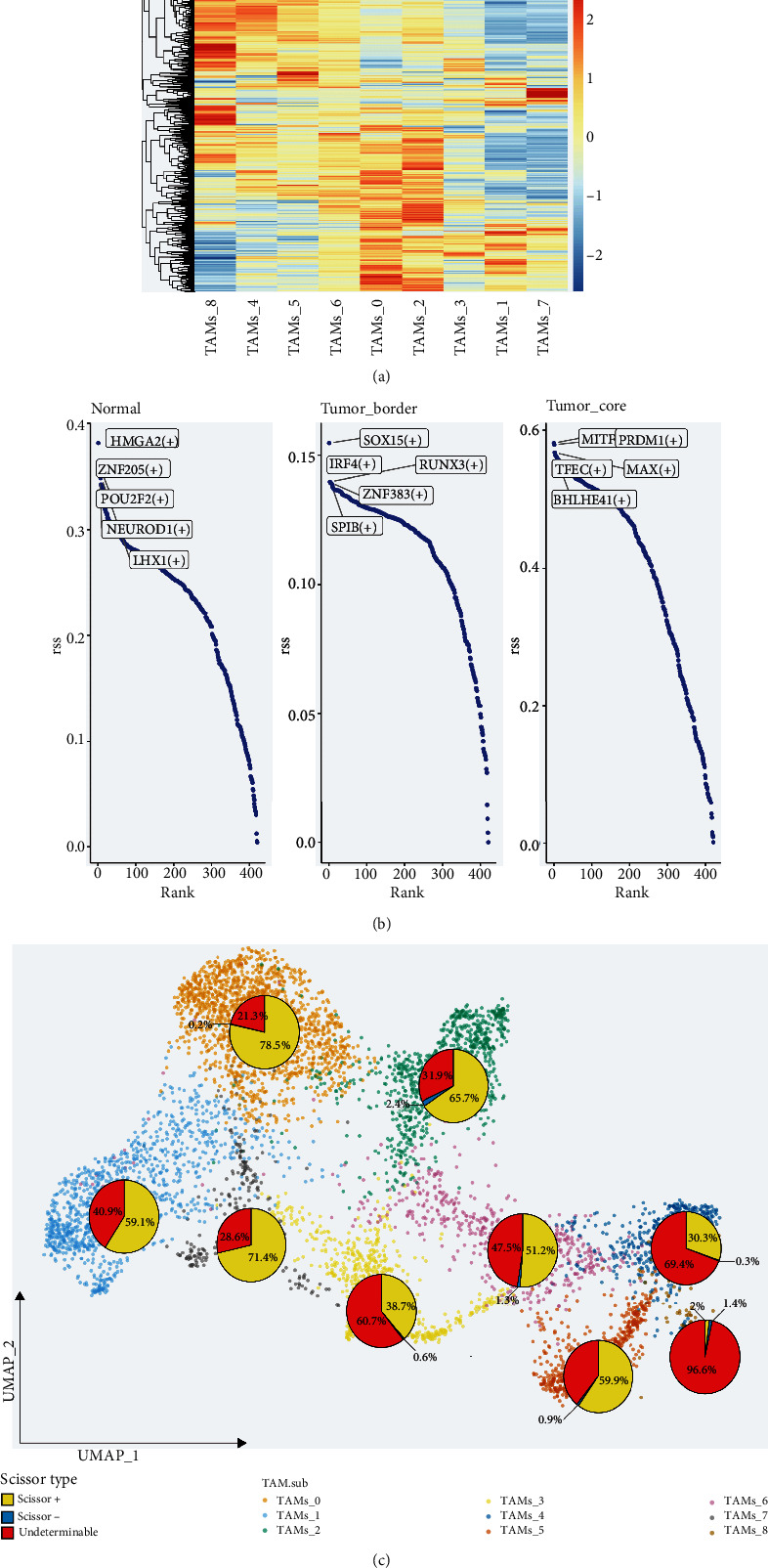
Gene regulatory network prediction and scissor analysis. (a) Heatmap of macrophage-related regulons in different subsets. (b) Top 5 regulons of macrophage in normal, tumor border, and tumor core. (c) Scissor analysis of macrophage subsets.

SCENIC analysis delineated the macrophage subsets into two distinct gene regulatory network (GRN) sets. TAMs_0, TAMs_2, TAMs_1, TAMs_3, and TAMs_7 exhibit GRN patterns aligning with the M2 phenotype, while the GRN patterns of the remaining four subsets notably diverge, resonating with the classical M1/M2 polarization paradigm (Figure 3(a)). The regulon specificity score (RSS) highlighted unique regulons across the three studied regions, with MITF(+), PRDM1(+), MAX(+), TFEC(+), and BHLHE41(+) emerging as the most specific regulons within the tumor core (Figure 3(b)). Scissor analysis (Figure 3(c)) further underscored the TAMs_0 subpopulation as the most prognostically significant for tumor patients, with 78.5% of its cells linked to poor prognosis, followed by TAMs_7 (71.4%) and TAMs_2 (65.7%).

### 3.4. Differentiation Trajectory of Macrophages and Pseudotime Genes

**Figure 4:**
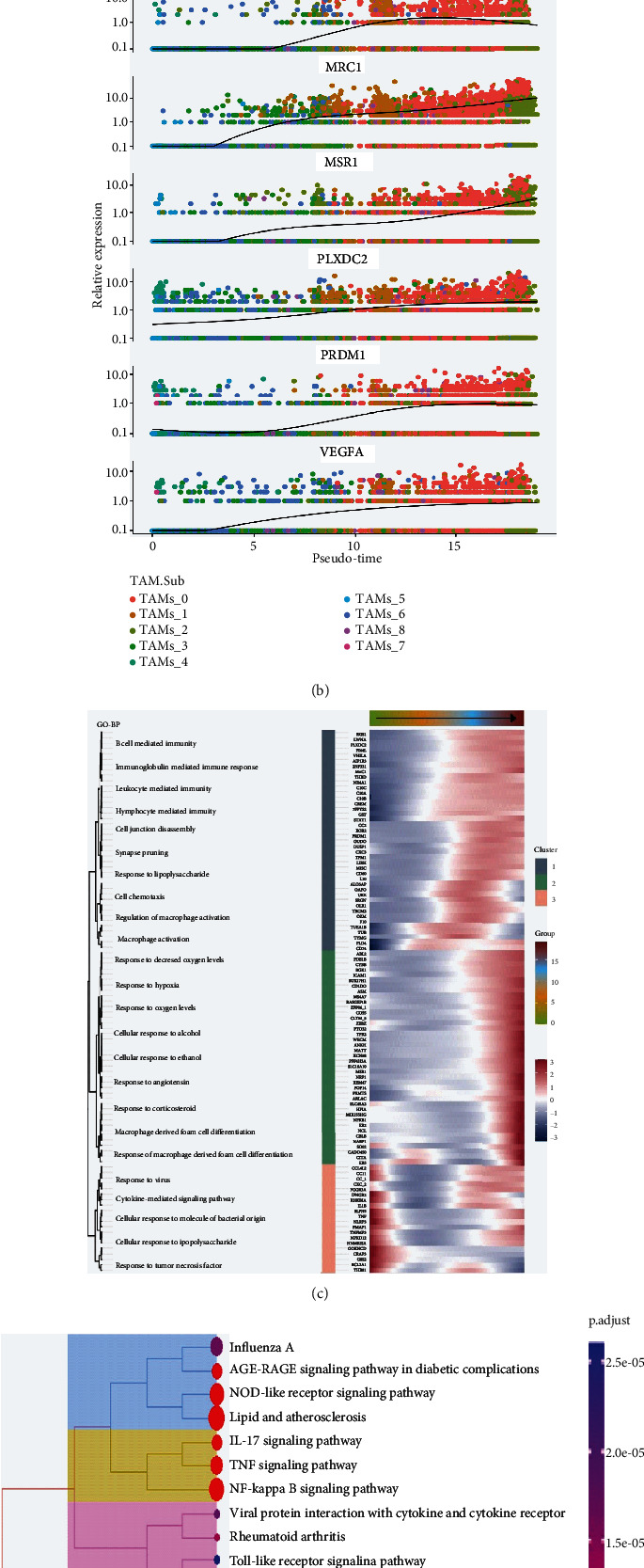
Trajectory analysis and genes for macrophage polarization. (a) Trajectory analysis of macrophage subsets. (b) Gene expression changes along pseudotime. (c) GO enrichment analysis of 101 pseudotime-related genes. (d) KEGG pathway analysis of cluster1 genes.

Utilizing Monocle2, we mapped the macrophage differentiation trajectory, revealing two distinct differentiation extremes during the pseudotime process. TAMs_4 and TAMs_5 initiated the trajectory, with subsequent appearances by TAMs_3, TAMs_6, TAMs_2, and TAMs_7, leading to TAMs_0 and TAMs_1 at the opposite extreme, with TAMs_0 playing a dominant role (Figure 4(a)). The intersection of pseudotime differentially expressed genes with the PRDM1(+) regulon identified 101 key pseudotime genes regulated by PRDM1 (Figure 4(b)). Enrichment analysis highlighted their involvement in crucial biological processes, including macrophage activation, leukocyte immune regulation, and response to hypoxia (Figure 4(c)). KEGG pathway analysis further implicated these genes in the NF-*κ*B, TNF, and IL-17 signaling pathways, underscoring their role in immune response and macrophage differentiation regulation (Figure 4(d)).

### 3.5. TCGA Data Analysis

**Figure 5:**
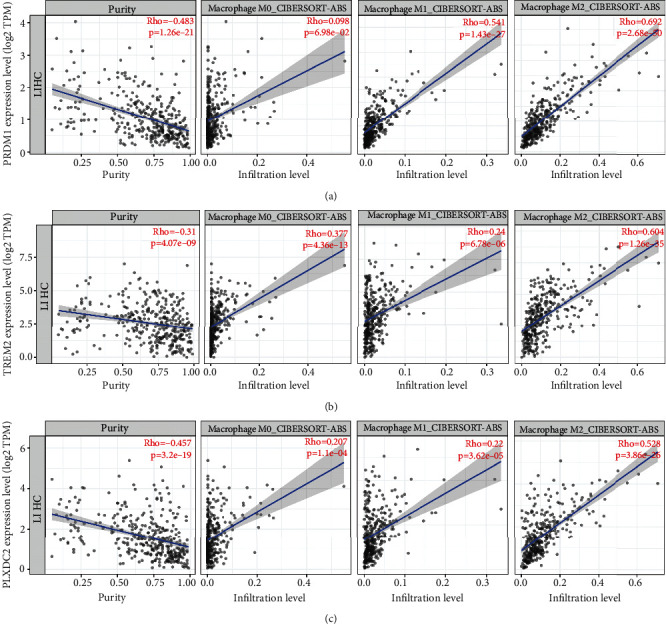
Correlation of gene expression with immune infiltration level of macrophage subsets. (a) Correlation of PRDM1 expression level with macrophage subsets. (b) Correlation of TREM2 expression level with macrophage subsets. (c) Correlation of PLXDC2 expression level with macrophage subsets.

TCGA data analysis reinforced a significant positive correlation between the PRDM1 gene and M2 macrophage infiltration, suggesting PRDM1 and 12 genes from cluster 1 as pivotal regulators of M2 macrophage polarization (Figure 5(a)). UALCAN database analysis revealed significant differences in these genes, correlating positively with poor tumor prognosis. TIMER2.0 database analysis pinpointed PRDM1 as having the strongest correlation with M2 macrophage infiltration (*r* = 0.691), followed by TREM2 (*r* = 0.604), PLXDC2 (*r* = 0.528), and CTSC (*r* = 0.516), highlighting their potential as biomarkers or therapeutic targets in HCC treatment strategies focused on modulating the tumor microenvironment (Figures 5(b) and 5(c)).

### 3.6. Expression of PRDM1 in HCC-Conditioned TAMs

**Figure 6:**
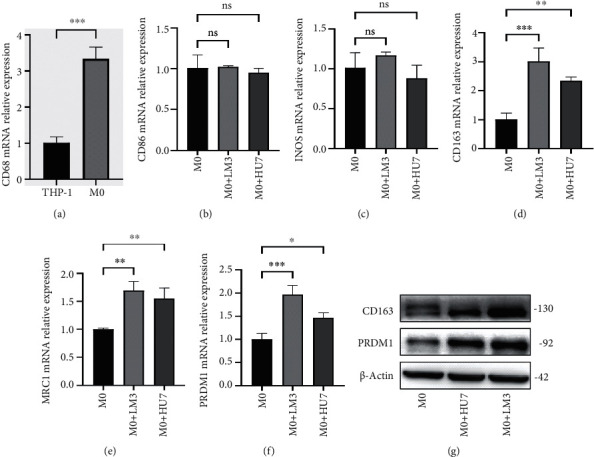
Expression of HCC-conditioned TAM markers. (a) Expression of panmacrophage markers. (b, c) Expression of M1 macrophage markers. (d, e) Expression of M2 macrophage markers. (f) The mRNA expression of PRDM1. (g) Protein expression of PRDM1 and CD163.

The transition of THP-1 cells into M0 macrophages was induced by culturing in a medium supplemented with 100 ng/mL PMA for 24 hours, resulting in a morphological shift from small, round, and translucent suspension cells to irregular, enlarged, and adherent macrophages. Subsequent RT-qPCR analysis highlighted a significant elevation in the expression of the macrophage marker CD68 in these differentiated cells compared to their THP-1 progenitors (*p* < 0.001), confirming successful macrophage differentiation (Figures 6(a), 6(b), and 6(c)).

Further, coculture of these M0 macrophages with hepatocellular carcinoma (HCC) cell lines for 72 hours not only increased cell volume but also notably altered the expression profile of macrophage markers. RT-qPCR results indicated a significant upregulation in the expression of M2 macrophage markers (MRC1, CD163) within HCC-conditioned TAMs, while the expression levels of M1 markers (CD86, iNOS) remained unchanged, suggesting a skew towards M2 polarization in the tumor microenvironment (Figures 6(d), 6(e), and 6(f)).

Western blot analysis provided corroborative evidence of this polarization, revealing significantly higher levels of CD163 and PRDM1 proteins in HCC-conditioned TAMs compared to M0 macrophages (Figure 6(g)). This data underscores the influence of HCC cell lines on macrophage polarization, specifically promoting an M2-like phenotype characterized by enhanced expression of PRDM1, a transcription factor implicated in the regulation of macrophage polarization and associated with the immunosuppressive tumor microenvironment.

## 4. Discussion

The efficacy of immune checkpoint inhibitors in advanced-stage liver cancer remains modest, a challenge intricately linked to the tumor's complex immunosuppressive microenvironment [26]. This complexity hinders the ability of current therapies to alleviate immunosuppression and bolster immune cell activity against cancer cells. Tumor-associated macrophages (TAMs), through their tumor-promoting mechanisms, offer numerous potential targets for immunotherapy, encompassing strategies to inhibit TAM recruitment, foster TAM depletion or apoptosis, enhance TAM phagocytosis, and steer TAM polarization towards antitumor phenotypes [27]. Yet, effectively targeting TAMs introduces several hurdles [28].

Our research illuminated the pronounced abundance of macrophages within the tumor microenvironment, especially in the tumor core, compared to adjacent and marginal areas, with a concurrent significant reduction in T cell proportions. Macrophage subgroups in the tumor core were found to express elevated levels of M2-like markers, implicating their role in fostering an immunosuppressive environment. Furthermore, these subgroups exhibited enhanced interactions with other cells, with M2-type signaling pathways, such as SPP1, markedly intensified within tumors. These findings suggest a reciprocal influence between the phenotype of tumor-associated macrophages and the tumor state, where each affects the polarization direction of the other.

The diversity in gene regulatory networks (GRNs) among different macrophage subgroups underscores TAMs' heterogeneity, aligning with trajectory analysis outcomes. Scissor analysis then highlighted the variable impact of these subgroups on tumor prognosis, with core area macrophage subgroups more likely associated with poor clinical outcomes. Interestingly, TAMs_4 and TAMs_5, leaning towards an M1-like phenotype, were still linked to unfavorable tumor prognoses in 30%-60% of cases. This finding challenges the conventional view of M1-like macrophages solely as tumor antagonists, suggesting that the dichotomy between M1 and M2 macrophages may not be as clear-cut, with the functional phenotype of mixed TAMs depending on a balance between macrophage states and the immune microenvironment [29].

Focusing on the core area, the PRDM1 regulon, notable for its high RAS and RSS, encompasses transcription factor PRDM1 and 644 potentially regulated target genes, including key M2-like markers. Further validation from TIMER2.0 database analysis confirmed significant correlations between PRDM1, its target genes, and M2 macrophage infiltration. The genes TREM2 and CTSC, known for their roles in M2 macrophage polarization in various tumors, highlight the complexity of TAMs' functions beyond traditional classifications [30–32].

Although PRDM1's regulatory role in macrophages warrants deeper exploration, its significant expression in M2-like macrophages within HCC-conditioned TAMs underscores its potential influence on TAM polarization and the immunosuppressive environment in HCC. Thus, PRDM1 emerges as a pivotal factor in directing TAM behavior, necessitating further experimental and clinical investigations to unravel its mechanisms and implications fully [33–35].

## 5. Conclusions

In summary, the transcription factor PRDM1 appears to be a critical regulator of TAM polarization towards an M2 phenotype, contributing to the immunosuppressive microenvironment in HCC. However, delineating its precise mechanisms and therapeutic potential in HCC requires more comprehensive experimental and clinical validation.

## Data Availability

Data will be available upon reasonable request.
